# Dr. Fisher

**Published:** 1897-12

**Authors:** 


### EDITORIAL DEPARTMENT.

F. E. DANIEL, M. D., Editor.
S. E. HUDSON, M. D., Managing Editor.
A. J. SMITH. M. D., Galveston, Associate Editor.

Published Monthly at Austin, Texas, by Drs. Daniel and Hudson. Subscription price $2.00 a year in advance.

Eastern Representatives: Messrs. Monihan & Fairchild, St. Paul Building, 220 Broadway, New York City.

Official organ of the West Texas Medical Association, the Houston District Medical Association, the Austin District Medical Society, the Brazos Valley Medical Association, the Galveston County Medical Society, and several others.

### DR. FISHER.

WE CALL ATTENTION to Dr. Fisher's reply to our remarks in the November issue and ask a careful reading of it. We disclaim any intention of doing the doctor injustice, and we beg to state that I immediately wrote Dr. Fisher to that effect, on having pointed out to me that I made a mistake with regard to the telegram, and that I wrote from recollection of conversation with State Health Officer Swearingen, and thus got the telegram and letter mixed. It was not a "reckless handling of the truth" (facts) but inattention to detail. I make now the statement that I was wrong in saying "Dr. Swearingen did not hear of the case until thirteen days after the *death* of the patient Torres." The statement in Dr. Fisher's telegram that "thirteen days" had "now elapsed"—since the inception—not death, misled me, as I wrote from memory only. As to the "indignities offered to Dr. Guiteras:" Will Dr. Fisher deny that when Dr. Guiteras asked permission to go to Galveston to investigate, he answered, emphatically, "No."? Dr. Allen Smith being called upon for what he knew of Dr. Guiteras, with whom he had been associated in the University of Pennsylvania, published a statement in the *Galveston News* in which he "felt ashamed and humiliated at the treatment accorded Dr. Guiteras by the Galveston profession." There were other items, such as making fun of his diagnosis after he left, and proposing, as I understand it, it was in the *News*, "to throw the mantle of charity on his errors." But it is not necessary to hunt up the matter, and I have forgotten further particulars. It may have been a member of the Council and not a member of the Board of Health who said Dr. Guiteras went to Galveston in the interest of New Orleans trade. But referring to Dr. Fisher's telegram to Dr. Swearingen, "for God's sake relieve us of this useless quarantine," is not that a pretty "loud protest against quarantining against suspicious cases?" Regarding "the highest medical authority, State and National," if it is not represented in the person of the head of the Health Department of Texas, and the representative of the U. S. Marine Hospital service, I do not know who would constitute it.

No, I do not "regard Dr. Allen J. Smith as an expert," tho' as to that matter I opine he has as good claims to it as some of the indiginous experts who figured at Galveston. But Dr. Smith being a skilled pathologist, with an excellent theoretical knowledge of yellow fever, his opinion is worthy of consideration; and as he isolated himself for fear of spreading the disease, I do think it would have been the part of prudence, and courtesy to him, respect for his opinion, to regard Torres' case as at least "suspicious," and to surround it with proper precautions; and under the circumstances, I do think it was the duty of a *subordinate officer* to report to his *chief*, the head of the department, the occurrence of a suspicious case. Dr. Fisher may know all about yellow fever (we don't pose as an expert ourself, but having practiced through the epidemic at Galveston in 1867 and made occasional autopsies, and having had charge of the sick at Lake, Mississipi, during the terrible epidemic of yellow fever there in 1878, and having treated the disease at Jackson and other places, I claim to know something of the disease myself, tho' I am not foisting my opinion on anybody), but Dr. Fisher must not forget that he is *subordinate* to the *State Health Officer* and bound to *obey his orders* in matters of contagious or infectious disease (see opinion of Attorney General Crane; see quarantine act), and it was clearly his duty to make the State Health Officer acquainted with the fact that Dr. Smith made an autopsy and thought the case was yellow fever; instead of pointedly omiting to answer the specific inquiry,—and should have treated it as a suspicious case. There is nothing inconsistent in what I said about the Surgeon General's inquiry if there was yellow fever in Galveston. Dr. Swearingen had no knowledge of the existence of any at the time, and so reported; Torres' case having been diagnosed (by Dr. Fisher, Smith to the contrary notwithstanding)—something else. But was it not something of a trick, shenanega—to say the board of health was unanimous—when the facts are that two members who were not "unanimous" resigned, to make it unanimous? The President: "How does the board stand on yellow fever, Mr. Secretary?" Secretary—"Four against and two for." "Ask their resignations and telegraph the governor that the board is unanimous." *

The fact of this whole matter is, Dr. Fisher's inordinate vanity,—"elephantiasis of egotism"—as Garfield said of Conklin, chafes and frets at any restraint; revolts at having to play second fiddle to *State* music; and now that it is all over he poses as the savior of his people, and makes a gallery play for effect. The editor *is* somewhat handicapped in writing of State quarantine, by the fact that he is an officer of the department; which fact Dr. Fisher throws up to him in a recent letter; "Your zeal for State quarantine is quite well understood," he says. To which we replied, "Certainly; I am an employe, and loyal to the administration; but that does not bias my views. I judge the tree by its fruits; we have a good system, as is evidenced by the long exemption of Texas from epidemics, while other states have suffered, despite the boasted advantages of a State board." Dr. Fisher ought to remember that there are those of us who clearly "understand" his "zeal" in fighting the State quarantine; there is something of the flavor of sour grapes in it. We "could a tale unfold" if so disposed; the animus is understood up this way.

---

Blanche Fray and Sweetheart are all barking at the quarantine department. Most of them who have assailed the management decry the expense to the State, and say that the quarantine was not effective, that it was never known to keep out epidemic disease; and in the next breath strenuously deny that there has been any yellow fever in Texas this year. If not; Swearingen, Guiteras, McLaughlin, Trueheart, Turner and other competent observers who pronounced yellow fever in Galveston and Hovston are mistaken, and every country doctor writing on the subject knows that he knew they *were* mistaken, in the name of consistency why did the disease not spread to Texas as it did to Mississippi, Lousiana, Alabama, Georgia and Tennessee? It all started from the same focus; Ocean Springs, where it got in from the Marine Hospital quarantine station at

---

* From S. W. Medical Record.

Ship Island through the carelessness of United States Marine Hospital service officials. Was it simply "the goodness and mercy of the Lord" that kept it out of Texas? or shall quarantine be given credit for just a little of it? (How about the "mercy of the Lord" as regards Edwards? and Boloxi and Scranton, New Orleans?) It looks like "anything to beat quarantine," true or not true.

---

BULLY: We are gratified to learn thro' Dr. E. J. Beall, of Fort Worth, who by the way writes "the Red Back is the brightest and most meritorious medical publication in Texas;" that the Fort Worth Medical School opens the session with one hundred and fifty matriculants.

---

AN ANONYMOUS editorial written in our Fort Worth Medical exchange, a new hand at the bellows who blows hot and cold simultaneously, says, speaking of the late epidemic of dengue that has prevailed in Southern Texas (Deriding Dr. Guiteras' opinion that there was any yellow fever in it, and decrying what he calls the useless interruption of commerce): "Our mathematics are too feeble to begin to approximate the cost to Texas," (of interrupted business). Our esteemed contemporary should give his mathematics some strengthening cordial, and set "them" to figuring on what *would have been the cost* to Texas, in loss or interruption of trade, had trade and travel *not* been interrupted when, and in the manner it was. It goes without saying, reasoning from past invasions and from the fate of sister states, that had not quarantine been instituted at Sabine river and elsewhere when it was, Texas would have suffered an invasion; and the "cost to Texas" would have been something more than the cost of quarantine and the diversion of the wheat [?] from Galveston, which this writer deplores. While his "mathematics" are in good working order, should they get over their feebleness, we suggest that he set them to work at figuring the "cost to Texas" of the epidemic of 1867. This writer referred to, calls Dr. Guiteras' diagnoses "a serious and irreparable slur upon the healthfulness of our beautiful and balmy gulf coast," as if Galveston had not been noted in former years for the deadly epidemics of yellow fever. Slur, indeed.

